# LTR retrotransposons shape genome architecture, function, and evolution in diverse plant species

**DOI:** 10.3389/fpls.2026.1813217

**Published:** 2026-07-30

**Authors:** Asmaa H. Hassan, Morad M. Mokhtar, Achraf El Allali

**Affiliations:** 1 Bioinformatics Laboratory, College of Computing, Mohammed VI Polytechnic University, Ben Guerir, Morocco; 2 Agricultural Genetic Engineering Research Institute, Agriculture Research Center, Giza, Egypt; 3 College of Chemical Sciences and Engineering, Chemical and Biochemical Sciences, Mohammed VI Polytechnic University, Ben Guerir, Morocco

**Keywords:** transposable elements, LTR- retrotransposons, LTR-RTs distributions, *Copia* and *Ty3-retrotransposons*, superfamilies, plant genome evolution

## Abstract

Long terminal repeat retrotransposons (LTR-RTs) are major components of plant genomes, shaping genome structure and evolution; however, their chromosomal distribution and lineage-specific dynamics remain incompletely understood. Here, we analyzed the abundance, spatial organization, and evolutionary history of intact LTR-RTs across 208 plant species using highquality chromosome-level genomes and the MegaLTR tool. Our analyses reveal extensive interspecific variability in LTR-RTs composition and chromosomal organization. Across families, LTR-RTs distributions are significantly non-random, with chromosome identity strongly influencing accumulation patterns. Although both *Copia* and *Ty3-retrotransposons* superfamilies exhibit significant inter-chromosomal heterogeneity, these superfamilies differ in the dimension of their non-randomness: *Copia* elements show more frequent heterogeneity between chromosomes, while *Ty3-retrotransposons* display stronger within-chromosome spatial structuring, consistent with preferential accumulation in recombination-suppressed heterochromatic regions. Distinct lineage patterns were observed, including highly structured *Ty3-retrotransposons* distributions in Poaceae. Insertion time analyses across 16 major clades revealed significant differences between genic and intergenic regions, with most clades showing younger insertions within genes. This pattern supports the role of purifying selection in removing older deleterious insertions from functional sequences. Together, these findings demonstrate that although regulatory mechanisms of LTR-RTs are broadly conserved, their chromosomal landscapes are shaped by superfamily-specific behavior, lineage history, and selective constraints, highlighting the central role of transposable elements in plant genome evolution.

## 1 Introduction

Transposable elements (TEs) are pieces of DNA that can move and replicate in the genomes of prokaryotic (Sawyer and Hartl, 1986) and eukaryotic organisms (Feschotte, 2008). TEs contribute to genome evolution in eukaryotic organisms by promoting genetic diversity and influencing gene expression, and may indirectly affect adaptation (Hassan et al., 2023). There are two main categories of transposons, classified by their replication mechanisms: class I TEs, also called retrotransposons, move through an RNA center using a copy-and-paste mechanism, while class II TEs move using a cut-and-paste mechanism by directly cutting and pasting DNA (Zhou et al., 2021).

Retrotransposons are divided into two main categories based on structure: Long Terminal Repeat (LTR-RTs) and non-LTR elements. LTR-RTs are characterized by two nearly identical LTR sequences flanked by target site duplications (TSDs). Their internal region encodes the Gag-Pol genes, which produce a polyprotein essential for RNA maturation, packaging, as well as protease, reverse transcriptase, RNase H, and integrase, which drive DNA synthesis and integration into the host genome. Two additional elements, the primer binding site (PBS) and the purine-rich polypurine tract (PPT), facilitate reverse transcription: the PBS provides the initiation site, while the PPT serves as a primer for DNA synthesis. LTR-RTs are further classified into *Ty3-retrotransposons* and *Copia* types, based on the sequence of the reverse transcriptase and integrase in the POL gene. Notably, some plant LTR-RTs encode an envelope-like protein, suggesting that they may function as endogenous plant retroviruses (Zhou et al., 2021). LTR-RTs significantly contribute to genome expansion in large-genome plants like maize, barley, and wheat, comprising 70–80% of their genomes (Bennetzen, 2005; Du et al., 2010).

The *Copia* and *Ty3-retrotransposons* superfamilies in plant genomes are further subdivided into different groups based on phylogenetic analyzes of their reverse transcriptase domains. *Copia* is subdivided into *Ale, Alesia, Lyco, Ikeros, Gymco I-IV, Bryco, Osser, TAR, Angela, Ivana, Tork, Retrofit, SIRE* and *Bianca*, while *Ty3-retrotransposons* is subdivided into *Tat-I-III, Athila, Galadriel, Reina, Chlamyvir, Retand, Tcn1, Tekay, Ogre, Phygy, Selgy* and *CRM* (Neumann et al., 2019; Mokhtar and El Allali, 2023).

Studies have shown that retroelements are distributed rather randomly across the genome. This hypothesis is supported by findings in *Sorghum bicolor*, where young LTR-RTs insertions (*<* 0.01 million years old) were reported to be broadly and apparently randomly distributed along chromosomes (Paterson et al., 2009). However, the frequently observed non-uniform distribution of TEs may result from the filtering out of transpositions within or near genes due to their deleterious effects, as well as the removal of LTR-RTs (or TEs in general) from highly recombining regions to minimize the risk of deleterious ectopic recombination (Manee et al., 2018; Zhang et al., 2011; Roze, 2023). Recent telomere-to-telomere assemblies have made the non-random chromosomal organization of LTR-RTs directly visible at base-pair resolution: in *Arabidopsis*, *Athila Ty3-retrotransposons* elements are concentrated at centromeres under H3K9me2 epigenetic control (Wlodzimierz et al., 2023; Naish et al., 2021), while in rice and maize, the proportions of centromeric *Ty3-retrotransposons* vary substantially among individual chromosomes of the same genome, from 0% to 74.3% across maize chromosomes alone, confirming that chromosome identity is a primary determinant of LTR-RT accumulation (Song et al., 2021; Chen et al., 2023).

Over the last two decades, the development of analytical tools for plant genomics has made it possible to better understand the distribution of TEs, particularly LTR-RTs, and to examine how their insertions contribute to variation among accessions. Increasing evidence indicates that the genomic distribution of TE families is not random. For example, a study of maize and tobacco identified nucleotide composition–based preferences for the insertion of five TE families, suggesting that each family targets specific genomic regions (Capel et al., 1993; Baucom et al., 2009). Similar findings have been reported in model plants such as *Arabidopsis thaliana* and *Oryza sativa*, where TE distribution correlates with other functional elements (Wu et al., 2006; Liu et al., 2014; Naito et al., 2009; Wei et al., 2016). At the chromosomal level, SORE-1 in soybean preferentially accumulate in euchromatic arms rather than pericentromeric regions (Nakashima et al., 2018), while in *Solanum lycopersicum*, full-length LTR-RTs of the *Ty3-retrotransposons* and *Copia* superfamilies also exhibit ordered distribution patterns (Paz et al., 2017).

Understanding why LTR-RTs are distributed non-randomly across plant chromosomes is important beyond the descriptive level; it has significant implications for interpreting plant genome evolution at a macroevolutionary scale. The non-random accumulation of LTR-RT families contributes to the enormous variation in genome size observed across flowering plants – a range spanning roughly two orders of magnitude. When a specific lineage undergoes a sudden burst of *Ty3-retrotransposons* or *Copia* amplification, its genome size can increase dramatically in a relatively short evolutionary window (Vitte and Panaud, 2005; Ammiraju et al., 2007). However, this expansion is not permanent: recombination-based processes, particularly solo-LTR formation through unequal homologous recombination, progressively erode retrotransposon content over time, creating a dynamic equilibrium between gain and loss that differs markedly among lineages (Xu et al., 2010; Liu et al., 2018; Zeng et al., 2017). Beyond genome size, lineagespecific amplifications of *Ty3-retrotransposons* and *Copia* families promote chromosomal rearrangements, influence centromere identity, and contribute to karyotype diversification. These processes are directly linked to speciation and adaptive evolution (Tao et al., 2025; Zhao and Ma, 2013; Cao et al., 2024; Souza et al., 2018). Insertion preferences of LTR-RT families also shape gene regulatory landscapes. Elements inserting near or within genes can modulate expression through epigenetic mechanisms, including RNAdirected DNA methylation (RdDM) and siRNA pathways, with lasting functional consequences for genome function and evolution (Papolu et al., 2022; Xu and Du, 2014). Additionally, as LTR-RTs accumulate and are removed at measurable rates, their distribution serves as a molecular record of past evolutionary events, including polyploidization, population bottlenecks, and shifts in selective pressure, making them powerful markers for reconstructing macroevolutionary histories (Vitte and Panaud, 2005; Ammiraju et al., 2007; Wang et al., 2025; Jiang et al., 2016; Li et al., 2022). Fully capturing these dynamics, however, requires exactly the kind of broad comparative framework we have applied. The forces that govern where retrotransposons end up including, selection against genic disruption, recombination-mediated removal, genetic drift in small populations, and the efficiency of host silencing pathways, vary considerably among plant families and cannot be reliably inferred from one or a handful of species (Li et al., 2022; Baidouri and Panaud, 2013).

Despite growing evidence that LTR-RTs distributions are non-random, the mechanisms governing their chromosomal organization across broad taxonomic scales remain poorly understood. Three interrelated questions motivate this study. First, whether inter-chromosomal heterogeneity in LTR-RTs density is a universal feature of plant genomes or reflects lineage-specific histories – a distinction that is biologically meaningful because chromosomes are not functionally equivalent; they differ in heterochromatin content, centromere size, recombination landscape, and gene density, all of which modulate where transposable elements can persist without incurring fitness costs (Feschotte, 2008; Bennetzen and Wang, 2014; Charlesworth et al., 1994). Second, whether the two major superfamilies, *Copia* and *Ty3-retrotransposons*, differ in chromosomal organization in ways that reflect their distinct integration preferences: *Ty3-retrotransposons* elements target chromodomain-containing integrase binding sites associated with heterochromatic histone marks (Gao et al., 2008; Kordiš, 2005), whereas *Copia* elements lack this mechanism and are predicted to show broader, more variable distributions (Gao et al., 2008; Quadrana et al., 2019). Third, we investigate whether insertion time distributions differ between genic and intergenic regions across LTR-RTs clades, a pattern that would provide direct evidence for purifying selection acting as a filter shaping contemporary chromosomal landscapes, consistent with a birth-and-death model of retrotransposon evolution (Lisch, 2013; Hollister and Gaut, 2009). To address these questions, we present a large-scale comparative analysis of 1,307,163 intact LTR-RTs across 208 chromosome-level plant genomes spanning 21 families. We apply a multi-test statistical framework to characterize chromosomal distribution patterns at multiple scales, with all p-values corrected for multiple testing. We test three explicit hypotheses: (H1) that LTR-RT chromosomal distributions are significantly non-random across plant lineages, with the degree of structuring varying in a lineage-dependent manner that reflects chromosome-specific genomic properties; (H2) that *Copia* and *Ty3-retrotransposons* superfamilies differ fundamentally in spatial coherence, with *Ty3-retrotransposons* showing stronger within-chromosome structuring consistent with preferential accumulation in recombination-suppressed heterochromatic compartments; and (H3) that insertion times are significantly younger within genes than in intergenic regions across most LTR-RT clades, supporting the role of purifying selection in continuously reshaping chromosomal LTR-RTs landscapes. These hypotheses frame our analysis as a comparative test of whether observed chromosomal distribution patterns are consistent with chromosomal architecture, superfamily biology, and selective constraint jointly determine where retrotransposons accumulate and persist in plant genomes.

## 2 Materials and methods

### 2.1 Plant genome sequences

Plant species with complete reference genomes, chromosome level assembly, and annotated sequences were retrieved from the NCBI (Sayers et al., 2021) and phytozome websites (Goodstein et al., 2012) accessed 30 November 2025. As the detection of intact LTR-RTs strongly depends on genome assembly quality, we selected high-quality chromosome-level genome assemblies for 208 plant species to minimize the impact of assembly quality on the results. These genomes represent 69 plant genera and cover a broad range of higher plant species (**Supplementary Table S1**). The selected genomes represent 21 plant taxonomic families: 4 Asteraceae, 6 Betulaceae, 12 Brassicaceae, 3 Cucurbitaceae, 17 Cyperaceae, 3 Dioscoreaceae, 12 Ericaceae, 35 Fabaceae, 13 Fagaceae, 3 Juglandaceae, 5 Lamiaceae, 12 Malvaceae, 7 Musaceae, 21 Poaceae, 2 Proteaceae, 20 Rosaceae, 4 Rubiaceae, 10 Salicaceae, 4 Sapindaceae, 6 Solanaceae, and 9 Vitaceae. The retrieved dataset of 208 plant genomes was analyzed to evaluate the evolution of LTR retrotransposons across diverse plant lineages (**Supplementary Figure 1**). This comprehensive dataset ensures reliable LTR-RTs analysis across different taxa and provides a robust framework for assessing the suitability of genome assemblies for various research applications. The species names, plant families, and genome data URLs for all genomes are listed in **Supplementary Table S1**. To evaluate the quality of the assembled plant genomes, we used the LTR Assembly Index (LAI) (Ou et al., 2018), a metric based on the identification and integrity of long terminal repeat retrotransposons (LTR-RTs). LAI scores were computed using the PlantLAI web server (Mokhtar et al., 2023a) (https://bioinformatics.um6p.ma/PlantLAI), and the results are presented in **Supplementary Table S1**. To ensure high assembly quality and reliable genomic representation, only plant genomes with an LAI score above 10 and high N50 were retained for downstream statistical analysis, resulting in a final dataset of 208 species.

### 2.2 Identification and annotation of intact LTR-RTs

The intact LTR-RTs were identified and annotated using the MegaLTR web server (Mokhtar and El Allali, 2023) with the following parameters: LTR size of 100–7000 bp, minimum and maximum distance between the two LTRs of 1000 and 15000 bp, minimum length of exact match pair 20, similarity of LTRs 85%, minimum coverage for protein domains in HMMScan 20, maximum E-value for protein domains in HMMScan 0.001, TE and HMM databases (rexdb), classification rule 80-80-80, the upstream and downstream distance of neighboring genes, 2000 bp, defines the window within which an LTR-RTs is classified as proximal to a gene; this parameter affects positional annotation only and does not influence LTR-RTs detection. To estimate the insertion time of LTR-RT elements, we conducted a comparative analysis of their 5^′^ and 3^′^ LTR sequences. As the two LTRs of LTR-RT are identical at the time of insertion and subsequently diverge through nucleotide substitution, the divergence between them serves as a molecular clock for dating insertion events. LTR divergence was quantified using the Kimura two parameter (K2P) model (Kimura, 1980), which calculates the number of substitutions per site (*K*) while accounting for differences between transition and transversion rates. The insertion time (*T*) was then calculated using the formula *T* = *K/*2*r*, where *r* is the single-lineage substitution rate (substitutions per site per year) and 2*r* represents the total divergence rate accumulated across both LTR lineages since insertion (SanMiguel et al., 1998). Following SanMiguel et al. (1998), who estimated the synonymous substitution rate for grass loci at *r* = 6.5 × 10^−9^ substitutions per site per year, we used a denominator of 2*r* = 1.3 × 10^−8^ for species in the Poales order. For all other plant species, we used *r* = 7.5 × 10^−9^, giving a denominator of 2*r* = 1.5 × 10^−8^ (Koch et al., 2000; González and Deyholos, 2012; Marcon et al., 2015; Mokhtar et al., 2023b). These values reflect commonly used approximations in plant genome studies and account for lineage-specific differences in substitution dynamics, particularly the elevated substitution rates observed in Poales (SanMiguel et al., 1998).

### 2.3 Statistical analysis of LTR-RTs and clade distribution

To analyze the distribution of LTR-RTs, the number of LTRs was counted in each window size, and the theoretical distribution was determined by dividing the total number of LTRs by the number of window sizes, assuming a uniform distribution. Different window sizes were calculated based on the genome sizes of the plants as previously defined by (Wu et al., 2006; Liu et al., 2014) (**Supplementary Table S2**). For each plant, an ANOVA test was performed to determine whether LTR densities differed significantly between chromosomes. To further investigate these differences, pairwise chromosome comparisons were performed using the Least Significant Difference (LSD) test. Additionally, a chi-square test was performed for each chromosome to assess whether the distribution of LTR-RTs follows a random pattern. Observed frequencies within each window size were compared with expected frequencies derived from the theoretical distribution. The degrees of freedom for each chromosome-level chi-square test were calculated as the number of windows on that chromosome minus one, such that df varied among chromosomes in proportion to their length (Wu et al., 2006; Liu et al., 2014). To further evaluate the randomness of LTR-RTs distributions and to ensure robustness against potential genomic biases, two complementary statistical approaches were applied: a Kolmogorov–Smirnov (KS) null model test and a permutation-based variance test. The KS test compared the empirical cumulative distribution of LTR-RTs positions along each chromosome with a theoretical uniform distribution representing the null hypothesis of randomness. This non-parametric test quantifies the maximum deviation between observed and expected distributions and provides a corresponding *p*-value to determine whether LTR-RTs deviate significantly from a uniform chromosomal distribution. In addition, a permutation variance test was conducted to assess the statistical significance of observed fluctuations in LTR-RTs density. Using a Monte Carlo framework, LTR-RTs positions were randomly permuted 10,000 times across the genome to generate a null distribution of variances under the assumption of random placement. The observed variance in LTR-RTs density for each species was then compared with this simulated distribution. A significant result (*p* ≤ 0.05) indicates that the observed clustering or dispersion of LTR-RTs cannot be attributed to random chance but instead reflects non-random genomic distribution patterns. Raw p-values obtained from the statistical tests were corrected for multiple comparisons across the 208 species using the Benjamini–Hochberg false discovery rate (FDR) procedure (Benjamini and Hochberg, 1995; Reiner et al., 2003). To test whether pairwise chromosome comparisons of LTR-RT density are more likely to be statistically significant when chromosomes differ more in size, we performed all pairwise chromosome comparisons within each of the 208 species for which chromosome-level assemblies with annotated chromosome lengths were available. Per-window LTR-RT density was compared between all chromosome pairs using two-sided Mann–Whitney U tests, selected over parametric alternatives due to the non-normal and zero-inflated distribution of TE-density windows. For each pair, the absolute chromosome size difference (|*s*_1_ − *s*_2_|, Mbp) was calculated from the master chromosome length reference table. To assess whether chromosome size disparity predicts statistical significance, two complementary approaches were used: (i) a Mann-Whitney U test comparing |size difference| between significant (*p <* 0.05) and non-significant chromosome pairs, and (ii) a logistic regression analysis modeling binary significance as a function of |size difference|, yielding an odds ratio per +1 Mbp of size disparity with its 95% confidence interval (Mann and Whitney, 1947; Hosmer et al., 2013). To complement statistical significance testing, effect sizes were calculated for all analyses. For one-way ANOVA tests, eta-squared (*η*^2^) was computed as a measure of the proportion of variance explained by genomic context. For Kolmogorov-Smirnov (KS) tests, the D-statistic was retained as a measure of effect size, representing the maximum difference between cumulative distributions. For Chi-square analyses, Cohen’s w was calculated to quantify the strength of association between observed and expected frequencies. For permutation-based variance tests, standardized effect sizes (Z-scores) were computed from the null distributions generated by random permutations (Hedges, 2008; Cankar and Bajec, 2003). Effect size estimates were reported alongside the corresponding test statistics in the **Supplementary Tables**.

For the clade distribution, the total number and percentage of each clade in each plant are calculated and the chi-square test is applied to determine if there are differences in the distribution of clades between plants. We calculated the mean insertion time for all clades in our group of plants and then examined the distribution of insertion times to determine the appropriate statistical test. Since the data did not meet the assumptions of parametric tests, we applied non-parametric tests to evaluate the correlation between insertion time and clade. Specifically, we used the Kruskal-Wallis test to determine whether there were significant differences in the distribution of insertion times between clades. Next, we calculated the insertion times of clades located inside and outside the genes for each plant.

## 3 Results

### 3.1 Comparative analysis of genome size and LTR-RTs content

**Figure 1:**
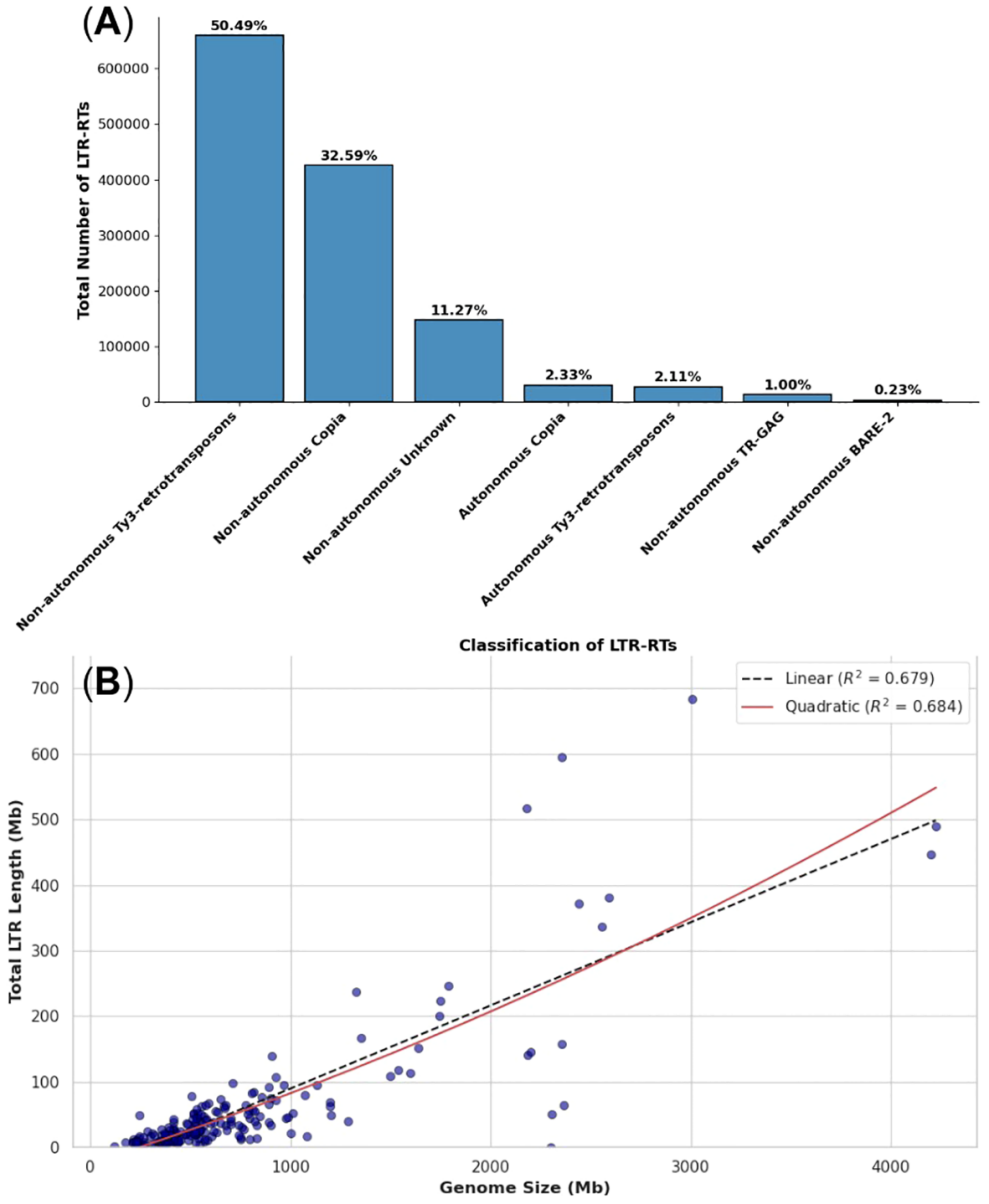
**(A)** Total numbers of intact LTR-RTs numbers in plants and **(B)** Correlation between Genome Size and Total LTR-RTs Content: This scatter plot shows a strong positive association between genome size and total LTR-RT length across 208 plant species. Both linear (*R*^2^ = 0.679) and quadratic (*R*^2^ = 0.684) models indicate consistent accumulation of LTR-RTs as genomes expand. The slight improvement in the quadratic fit suggests a subtle non-linearity, with a steeper increase in LTR-RT content at larger genome sizes. High-density outliers, such as *L. perenne*, are also observed.

The analysis of 208 plant genomes identified a total of 1,307,163 intact LTR-RTs. The vast majority of these elements were classified as non-autonomous, accounting for 95.57% (1,249,266 elements) of the total population, while autonomous elements represented only 4.43% (57,937 elements). Among non-autonomous elements, *Ty3-retrotransposons* were the most abundant superfamily, comprising 659,939 elements, equivalent to 50.49% of the total LTR-RT pool and 52.83% of all non-autonomous elements. The *Copia* superfamily followed, representing 32.59% (425,940 elements) of all identified LTR-RTs. Other non-autonomous categories included *TR-GAG* (1%), *BARE-2* (0.23%), and a substantial proportion of unclassified “Unknown” elements (11.27%; 147,345 elements) highlights a current limitation of LTR-RT classification frameworks, particularly in less-studied plant lineages, suggesting that a significant fraction of plant LTR-RT diversity remains phylogenetically uncharacterized (**Figure 1A**).

The assignment of genome size to LTR-RTs content revealed substantial variation among species. The genomes examined range from 119.1 Mbp in *A. thaliana* to 4225.6 Mbp in *H. vulgare*. Accordingly, LTR-RTs content also varies widely, with high percentages observed in species such as *L. perenne* (25.3%), *Z. mays* (23.7%) and *H. annuus* (22.7%), likely reflecting their contribution to genome expansion. In contrast, species such as *L. perrieri* (0.4%), *B. oleracea* (0.9%) and members of the *Gossypium* genus (e.g., *G. rotundifolium*) have minimal LTR-RTs content, suggesting more efficient repression mechanisms (**Supplementary Table S3**). Most species fall within the 1–6% LTR-RTs range, indicating a largely conserved pattern.

Correlation analyses revealed a moderate positive relationship between genome size (Mb) and the percentage of LTR-RTs. In contrast, a stronger association was observed between genome size and absolute LTR-RTs content. Both the linear and quadratic models provided a moderate positive relationship between genome size and the length of LTR-RTs (*R*^2^ = 0.679 vs. 0.684), indicating a non-linear increase in LTR-RTs content with increasing genome size. These results indicate that larger plant genomes tend to accumulate greater quantities of LTR-RTs sequence overall, although the proportional contribution (percentage) varies among species (**Figure 1B**). Notably, there are exceptions to this trend, highlighting the complexity of LTR-RTs influence. Despite similar genome sizes, *P. persica* and *P. dulcis* (227.4 and 227.6 Mbp, respectively) differ significantly in the number of LTR-RTs. In addition, *G. arboreum* has a relatively low proportion of LTR-RTs despite its large genome.

The structural landscape of LTR-RTs was dominated by non-autonomous elements, which accounted for approximately 95.57% of the total LTRs across the dataset. Among these, non-autonomous *Ty3-retrotransposons* elements were the most abundant, representing an average of 52.83% of the non-autonomous fraction, followed by non-autonomous *Copia* elements at 34.1%. This pattern was particularly evident in *A. tauschii*, where non-autonomous *Ty3-retrotransposons* and *Copia* elements comprised 55.75% and 29.21% of the LTR fraction, respectively.

Autonomous elements were comparatively less abundant, although their relative contribution varied among species. Their proportional contribution ranged from 0.2% in *P. pruinosa* to 18.9% in *S. sclarea*, with most species falling within a moderate range of 2–8%. Elevated autonomous fractions in species such as *Z. mays* and *S. sclarea* suggest lineage-specific differences in transpositional potential, whereas minimal contributions in species including *P. pruinosa* and *P. coccineus* indicate tighter regulatory control or reduced recent activity. In contrast, certain species displayed comparatively higher proportions of specific autonomous superfamilies. In *Z. mays*, 13.7% of intact LTR-RTs were classified as autonomous *Copia* elements and a further 2.6% as autonomous *Ty3-retrotransposons*, together accounting for its exceptionally high total autonomous fraction of 16.3% and reflecting substantial structural variation among species (**Supplementary Table S3**).

Unclassified LTR-RTs also contribute significantly to genomic diversity. In *L. perenne* and *L. rigidum*, unknown elements account for 30.12% and 33% of LTR-RTs, respectively (**Supplementary Table S3**).

### 3.2 Statistical analysis of LTR-RTs distribution

#### 3.2.1 Quantifying inter-chromosomal heterogeneity and intra-chromosomal clustering of LTR-RTs

**Figure 2:**
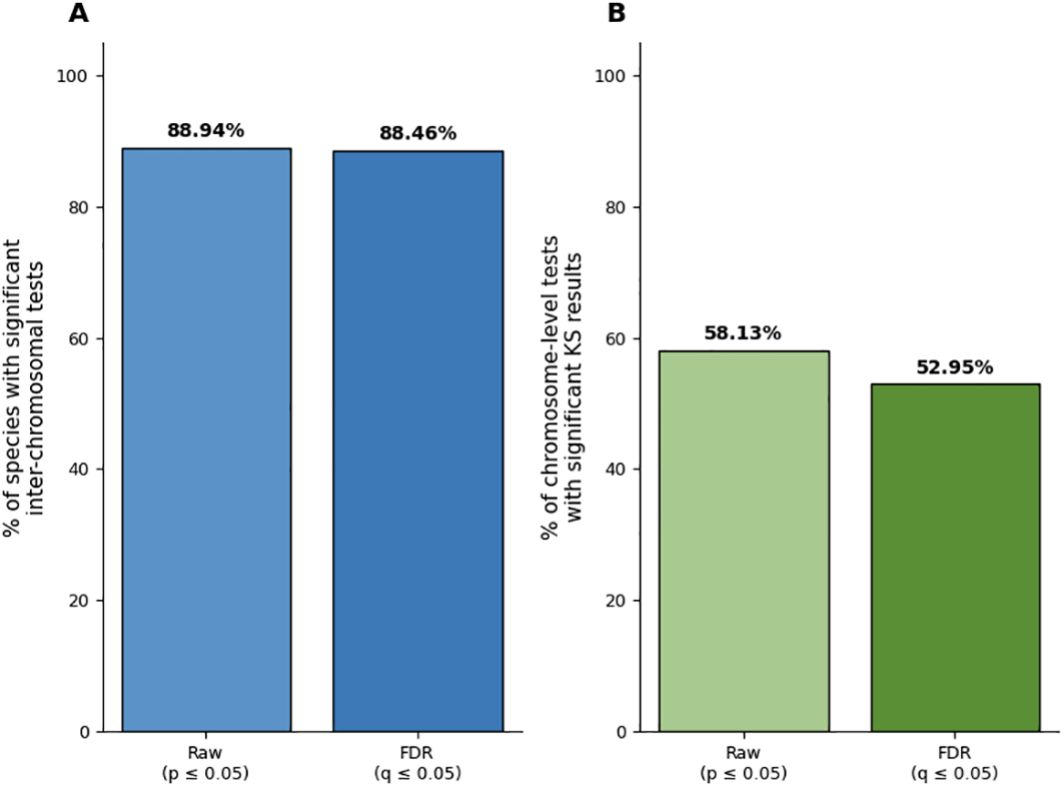
Robustness of LTR-RTs non-random distribution to multiple-testing correction. **(A)** Percentage of the 208 analyzed plant species showing significant inter-chromosomal heterogeneity in LTR-RT density based on ANOVA tests using raw and FDR-corrected *p*-values. **(B)** Percentage of significant chromosome-level Kolmogorov–Smirnov (KS) tests assessing within-chromosome non-randomness across the analyzed chromosomes. The high proportion of significant results after correction confirms that LTR-RT distributions are strongly non-random across plant genomes.

To analyze the distribution of LTR-RTs along the chromosomes in each plant genome, the number of LTR-RTs was calculated using window sizes determined for each chromosome. ANOVA tests were conducted to statistically assess the heterogeneity of LTR-RTs distributions between chromosomes for each plant. Raw *p*-values were corrected for multiple testing using the Benjamini–Hochberg false discovery rate (FDR) procedure; both raw and adjusted values are reported in **Supplementary Table S4**. Our analysis of 208 plant species revealed that approximately 88.94% of species had ANOVA *p*-values ≤ 0.05, and 88.46% remained significant after FDR correction, indicating that LTR-RTs density is not uniform across the genome but varies significantly among different chromosomes (**Figure 2**). In contrast, roughly 11.06% of the species exhibited *p*-values *>* 0.05, suggesting a more balanced distribution of LTR-RTs across their chromosomal sets. Effect sizes (*η*^2^) ranged from 0.011 to 0.997 (median = 0.133), indicating that chromosome identity explained a moderate proportion of within-genome variation in LTR-retrotransposon density. Approximately 48.1% of species exhibited large effects (*η*^2^ ≥ 0.14), demonstrating that the observed inter-chromosomal heterogeneity was not only statistically significant but also biologically meaningful (**Supplementary Table S4**). A large proportion of species showed highly significant results (*p <* 0.001), reflecting pronounced inter-chromosomal variation in LTR-RTs density. Many species displayed extremely strong heterogeneity, with exceptionally low ANOVA *p*-values, including *R. rugosa* (*p <* 0.001), *V. sativa* (*p <* 0.001), *M. micrantha* (*p <* 0.001), *N. attenuata* (*p <* 0.001), and *Q. lobata* (*p <* 0.001), indicating pronounced differences in LTR-RTs density among chromosomes. Strong but less extreme inter-chromosomal variation was also common, as seen in species such as *R. canina* (*p <* 0.001), *P. tomentosa* (*p <* 0.001), *C. rostrata* (*p <* 0.001), and *Q. alba* (*p <* 0.001). Moderate yet statistically significant differences were observed in several species, including *T. striatum* (*p* = 0.052). Note that some species previously considered significant in preliminary assessments, such as *C. hispida* (*p* = 0.191) and *A. thaliana* (*p* = 0.073), showed *p*-values above the conventional 0.05 threshold in this dataset. In contrast, a subset of species showed no significant inter-chromosomal differences in LTR-RTs density, such as *C. melo* (*p >* 0.05) and *B. variegata* (*p >* 0.05), suggesting relatively uniform chromosomal distributions. For several species, including *C. depauperata* (*p <* 0.001), *C. colurna* (*p <* 0.001), *C. nigra* (*p <* 0.001), and *G. max* (*p <* 0.001), ANOVA *p*-values were exceptionally low, indicating profound inter-chromosomal heterogeneity.

To identify specific chromosome pairs contributing to inter-chromosomal variation in LTR-RTs density, *post-hoc* pairwise comparisons with FDR correction were performed. Across all analyzed species, a total of 24,808 pairwise chromosome comparisons were conducted (**Supplementary Table S5**). After FDR correction, significant differences in LTR-RTs density were detected in 27.3% of these comparisons (*p <* 0.05), while the majority (72.7%) showed no significant differences (*p >* 0.05), indicating that although many chromosomes within a genome maintain comparable LTR-RTs densities, a substantial fraction exhibits significant variation. The degree of inter-chromosomal differentiation varied markedly among taxa. High levels of differentiation were observed in *Z. mays* (80%) and *A. tauschii* (66.7%), whereas intermediate levels were detected within the *Acer* genus, including *A. campestre* (45.5%) and *A. yangbiense* (41.0%). In contrast, members of the *Vitis* genus, such as *V. wilsoniae* (10.5%) and *V. yunnanensis* (14.6%), exhibited relatively low proportions of significant pairwise differences. Using *C. sativus* as an example, significant differences were confined to specific chromosome pairs, such as chr1 versus chr3 and chr6, whereas other comparisons, including chr1 versus chr4, showed no significant differences.

To confirm that the observed patterns were not statistical artifacts, Kolmogorov–Smirnov (KS) nullmodel and permutation-based variance tests were applied as robustness checks; full results are provided in **Supplementary Tables S6, S7**. These analyses confirmed that 52.95% of chromosomes exhibited non-random LTR-RT distributions according to the KS test (FDR-corrected, *p <* 0.05) (**Figure 2**, while 42.11% of chromosomes showed significant inter-chromosomal variance in the permutation-based tests (FDR-corrected, *p <* 0.05). ANOVA was designated as the primary statistical test because it tests between-chromosome differences in mean density, the hypothesis of biological interest, and is not inflated by the large total sample size in the way that omnibus variance and KS tests are. The KS null-model and permutation-based variance tests are retained as secondary robustness analyses. These findings confirm that the spatial arrangement of LTR-RTs within chromosomes is non-random, even in species where global density variation appears relatively subtle (e.g., *F. vesca*).

Chi-square tests, which evaluate local deviations from a uniform expectation, were also applied as additional validation; 59.96% of the 2,937 chromosome-level tests were significant at *p <* 0.05 (**Supplementary Table S8**). Taken together, these complementary approaches consistently support the conclusion that LTR-RT distributions are non-random across all studied species.

Across 208 species, a total of 24,808 pairwise chromosome comparisons were performed, of which 7,335 (29.57%) were statistically significant after FDR correction(*p <* 0.05). Significant pairs had a median absolute chromosome size difference of 11.78 Mbp, more than twice that of non-significant pairs (4.15 Mbp; Mann–Whitney *U* = 95,224,507.5, *p <* 2.2 × 10^−16^; **Supplementary Table S9**). Logistic regression confirmed that chromosome size disparity significantly predicts the probability of a significant LTR-RTs density difference (*β* = 0.102, *p <* 2.2 × 10^−16^; odds ratio per +1 Mbp = 1.107, 95% CI 1.103–1.111; pseudo-*R*^2^ = 0.17; **Supplementary Figure 2**). Each additional Mbp of chromosome size disparity increases the odds of a significant pairwise comparison by approximately 10.7%. However, the modest pseudo-*R*^2^ value (0.17) indicates that chromosome size disparity accounts for only a limited proportion of the observed LTR-RTs density heterogeneity, confirming that chromosome size is a real but partial confounding factor rather than the primary driver of chromosome-level variation in LTR-RTs density.

#### 3.2.2 Chromosomal patterning of LTR-RTs across plant families

After a comprehensive analysis, we filtered the dataset to focus on the ten most species-rich plant families. We then evaluated the chromosomal distribution of LTR-RTs across these families using various statistical approaches, including ANOVA, *Post hoc* LSD test, Chi-square tests, permutation-based variance tests, and Kolmogorov–Smirnov (KS) tests against a null model (**Supplementary Table S10**).

The ANOVA results after FDR correction revealed significant heterogeneity in LTR distribution among chromosomes for most families. Strong signals were detected in Salicaceae, Fagaceae, Poaceae, Cyperaceae, Malvaceae, Rosaceae, Fabaceae, and Vitaceae where ≥ 88% of tests were statistically significant (*p* ≤ 0.05). In contrast, Brassicaceae, and Ericaceae exhibited weaker, though still notable, patterns, with approximately 68.9–66.7% of tests reaching significance, suggesting moderate levels of inter-chromosomal variation in these groups.

*Post hoc* LSD tests further identified significant chromosomal biases in LTR distributions across all families when both superfamilies were analyzed jointly (**Supplementary Table S10**). though the strength of these signals varied. The highest proportions of significant results were observed in Fagaceae (53.25%), Malvaceae (42.07%), and Poaceae (41.81%), followed by Rosaceae (30.72%), Brassicaceae (29.86%), Fabaceae (29.51%), and Salicaceae (27.16%). In contrast, Cyperaceae (20.23%), Vitaceae (18.81%), and Ericaceae (12.07%) exhibited the lowest proportions of significant pairs, suggesting a comparatively uniform LTR distribution across chromosomes in that lineage.

Additional statistical tests (Chi-square, Kolmogorov–Smirnov, and permutation-based variance tests) were used to assess robustness. These analyses supported non-random LTR-RT distributions across all families, with permutation and KS tests showing highly significant deviations from random expectations (**Supplementary Table S10**).

#### 3.2.3 Chromosomal distribution patterns of *Copia* and *Ty3-retrotransposons* superfamilies

*Copia* and *Ty3-retrotransposons* show quantifiable but modest differences in chromosomal organization, which are predicted to produce distinct chromosomal distribution patterns. *Ty3-retrotransposons* encode a chromodomain within their integrase that directly binds heterochromatic histone marks (H3K9me2/me3), directing insertions preferentially into pericentromeric and heterochromatic regions (Gao et al., 2008; Kordiš, 2005). *Copia* elements lack this chromodomain-based targeting mechanism and are therefore predicted to show broader, more dispersed, and lineage-variable chromosomal distributions (Gao et al., 2008; Quadrana et al., 2019). We tested whether these mechanistic differences produce detectable differences in chromosomal organization across our dataset of 208 plant species. To assess whether the chromosomal organization of LTR retrotransposons differs between the two major superfamilies, *Copia* and *Ty3-retrotransposons*, we applied ANOVA as the primary test, complemented by *post-hoc* pairwise comparisons. Chi-square, Kolmogorov–Smirnov (KS), and permutation-based variance tests were retained as robustness analyses in the **Supplementary Materials**. All p-values were corrected for multiple testing using the Benjamini–Hochberg false discovery rate (FDR) procedure, with Bonferroni correction applied as a conservative confirmation.

ANOVA revealed significant inter-chromosomal variation in LTR-RT density for the large majority of species in both superfamilies. After FDR correction, 88.46% of species showed significant interchromosomal heterogeneity for *Copia* elements, and 84.62% of species for *Ty3-retrotransposons* elements, median effect sizes were highly similar (*η*^2^ = 0.080 and 0.084, respectively), indicating comparable levels of inter-chromosomal heterogeneity. (**Supplementary Table S11**, **Supplementary Figure 3**). Under the more conservative Bonferroni correction, 59.62% and 62.98% of species remained significant for *Copia* and *Ty3-retrotransposons*, respectively.

To identify the specific chromosome pairs driving these global patterns, we conducted *post hoc* LSD pairwise comparisons. Significant differences (FDR-adjusted *q* ≤ 0.05) were detected in 12.89% of pairwise comparisons for *Copia* and 11.47% for *Ty3-retrotransposons* (**Supplementary Table S12**, **Supplementary Figure 3**), consistent with the slightly broader inter-chromosomal variability observed for *Copia* at the genome-wide level.

Chromosome-level Chi-square tests further confirmed non-random distributions. Overall, Ty3retrotransposons elements showed a higher proportion of significant Chi-square results after FDR correction (approximately 55.02%) compared to *Copia* (approximately 44.02%), indicating more consistent chromosomal biases for *Ty3-retrotransposons* (**Supplementary Table S13**, **Supplementary Figure 3**).

To assess whether these patterns represented extreme departures from random expectations, we applied permutation-based variance tests. The low proportion of significant permutation tests (approximately 32.4% for *Copia* and 34.8% for *Ty3-retrotransposons*, FDR-corrected). This indicates that, when assessed at the superfamily level, the observed within-chromosome variance in LTR-RT density frequently exceeds what would be expected under random placement. This contrasts with the near-universal significance in the pooled analysis, which is substantially inflated by the combined sample size (n = 1,273,577) and therefore cannot be taken as independent evidence of per-superfamily non-randomness. ANOVA, which tests for between-chromosome heterogeneity rather than global variance, remains our primary statistical framework; effect sizes are reported alongside (**Supplementary Table S14**, **Supplementary Figure 3**).

Finally, Kolmogorov–Smirnov (KS) tests revealed significant deviations from the null model of random placement in 53.29% of chromosome-level tests for *Ty3-retrotransposons*, compared with 35.81% for *Copia* after FDR correction, a difference of approximately 17 percentage points. While both superfamilies deviate from random placement in a substantial proportion of chromosomes, both values indicate that within-chromosome spatial structuring deviates from random expectation in a substantial proportion of chromosomes. This suggests that non-random clustering is a pervasive feature of these superfamilies at the per-chromosome level, a finding that should be interpreted in conjunction with ANOVA effect sizes (**Supplementary Table S15**, **Supplementary Figure 3**).

Together, these analyses reveal two distinct dimensions of non-randomness. *Copia* elements are more frequently heterogeneous between chromosomes, reflecting a more dispersed, lineage-specific accumulation pattern. In contrast, Ty3 elements show stronger and more coherent within-chromosome spatial structuring, consistent with their well-documented preferential accumulation in recombination-suppressed pericentromeric and heterochromatic regions.

Having established that *Copia* and *Ty3-retrotransposons* differ in their genome-wide chromosomal organization across 208 species, we next asked whether these superfamily-specific patterns are conserved across plant lineages or vary among families. To address this, we applied our full statistical framework to the *Copia* and *Ty3-retrotransposons* superfamilies separately across the ten most species-rich plant families (**Supplementary Table S16**).

Across families, ANOVA results after FDR correction consistently revealed significant inter-chromosomal heterogeneity for both superfamilies, indicating that chromosome identity strongly influences LTR-RTs distribution. In several families, such as Fagaceae (100% for both superfamilies) and Poaceae (95.24% *Copia,*100% *Ty3-retrotransposons*), and Salicaceae (100.00% Copia, 90.00% *Ty3-retrotransposons*), ANOVA results showed particularly strong signals, reflecting pronounced chromosomal structuring. In contrast, families such as Brassicaceae (58.33% Copia, 75.00% *Ty3-retrotransposons*) and Ericaceae (66.67% Copia, 58.33% *Ty3-retrotransposons*) exhibited weaker but still detectable heterogeneity.

*Post hoc* LSD pairwise comparisons showed that significant differences were often restricted to specific chromosome subsets. In most families, *Ty3-retrotransposons* elements had a higher proportion of significant pairwise contrasts than *Copia*, most notably Fagaceae (15.38% *Copia,* 48.60% *Ty3-retrotransposons*) and Poaceae (15.00% *Copia, *24.4% *Ty3-retrotransposons*). However, exceptions like Malvaceae (29.90% *Copia, *18.60% *Ty3-retrotransposons*) and Brassicaceae (23.60% *Copia, *9.00% *Ty3-retrotransposons*) demonstrate stronger localized chromosomal biases for *Ty3-retrotransposons*. In Fabaceae, the difference between superfamilies was relatively narrow (16.00% *Copia, *17.83% *Ty3-retrotransposons*), suggesting limited superfamily-specific chromosomal divergence in this lineage.

Additional statistical tests, including Chi-square, Kolmogorov–Smirnov (KS), and permutation-based variance tests, were used to assess the robustness of LTR-RT distribution patterns. These analyses supported non-random chromosomal organization across all families, with permutation and KS tests showing highly significant deviations from random expectations (**Supplementary Table S16**). While both superfamilies exhibit pervasive non-random structuring, *Ty3-retrotransposons* generally show more coherent chromosomal integration, whereas *Copia* elements display greater spatial variability influenced by lineage-specific genome architecture.

### 3.3 Relation between clade and insertion time

**Figure 3:**
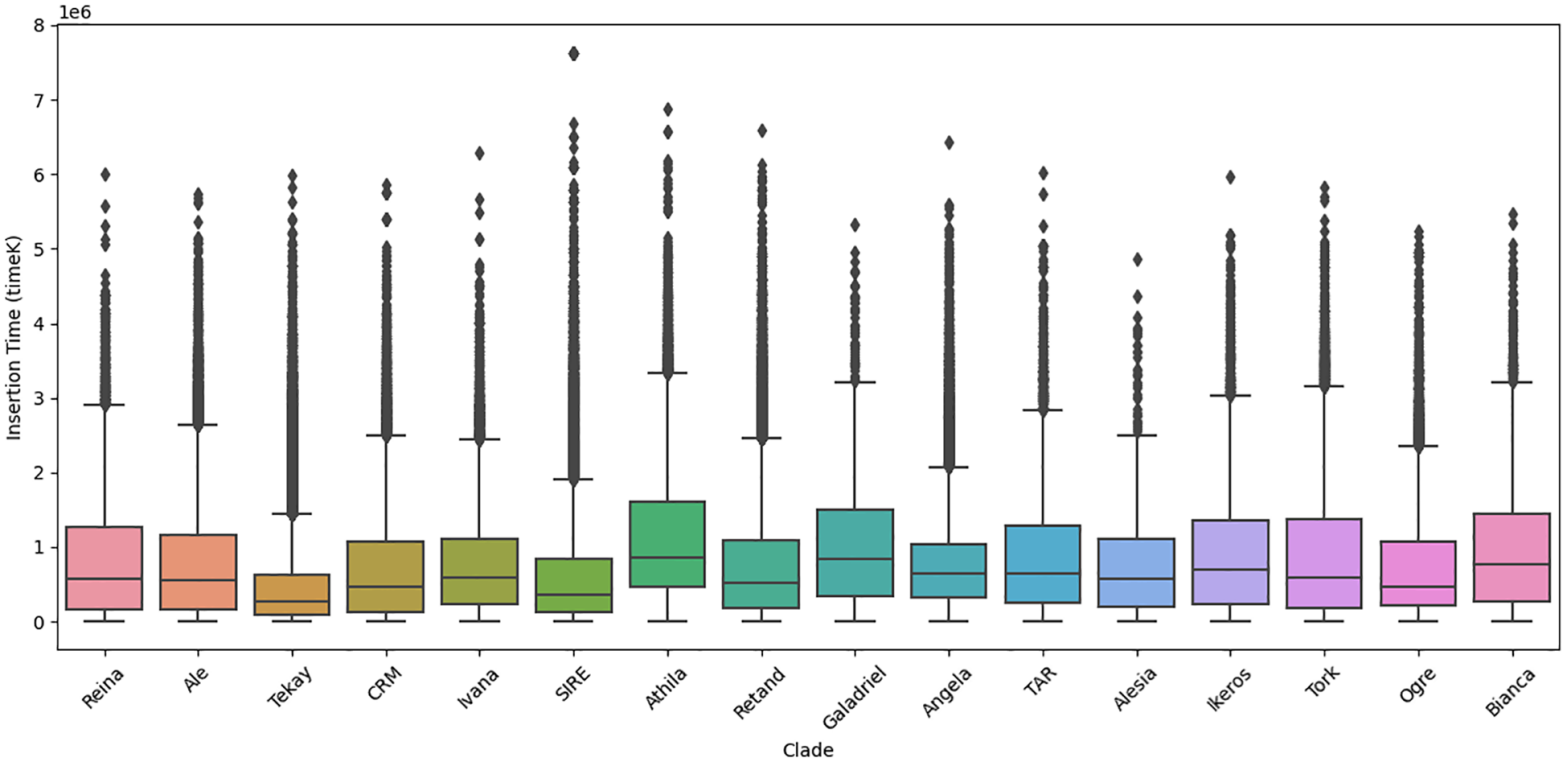
Distribution of LTR-RTs insertion times (*timeK* in thousand years) across different clades.

To evaluate whether landscape of LTR-RT insertion times differed significantly among clades, we examined the full distribution of divergence times rather than relying solely on mean values. we applied the Kruskal–Wallis test, as insertion times were not normally distributed. The test yielded a significant result (H = 50.28, p = 0.0001), confirming that divergence times differ significantly between clades. This finding indicates that clade identity influences insertion dynamics, potentially reflecting lineage-specific evolutionary or genomic factors (**Figure 3**). Divergence time landscapes (**Figure 3**) reveal that recently active clades such as *Tekay* and *SIRE* display strongly right-skewed distributions with a high density of recent insertions, whereas older clades such as *Athila* and *Galadriel* show broader, flatter distributions consistent with a longer and more heterogeneous accumulation history. Intermediate clades (*Ale*, *Angela*, *Ivana*) display unimodal distributions centered near the median insertion time.

Consistent with this statistical result, mean insertion times corroborate these distributional patterns and reveal a clear spectrum of activity (*timeK*, in thousand years (Kyr)) vary considerably between the different clades, revealing a clear spectrum of activity: *Tekay* (461 Kyr) and *SIRE* (576 Kyr) exhibit the most recent mean insertion times, indicating relatively recent activity, while *Athila* (1,060 Kyr) and *Galadriel* (1,030 Kyr) exhibit the oldest mean insertion times, suggesting the persistence of older elements. Also, intermediate clades such as *Ale* (767 Kyr), *Angela* (767 Kyr) and *Ivana* (760 Kyr) are close to the median, while *Alesia* (838 Kyr), *Tork* (892 Kyr), and *TAR* (888 Kyr) show a slower but variable timing (Mean insertion times cited here represent the combined average across genic and intergenic insertions; values by genomic location are reported in **Table 1**). These patterns may reflect clade-specific differences in genomic dynamics. The younger mean genic insertion times observed in faster clades such as *Tekay* and *SIRE* are consistent with either preferential insertion near genes or more rapid purging of older genic insertions by purifying selection – mechanisms that cannot be distinguished from insertion time data alone. By contrast, the older and more broadly distributed insertion times of clades such as *Athila* are consistent with long-term retention in heterochromatic domains, as previously reported.

**Table 1:** Comparative analysis of mean insertion times for LTR-RT clades inside and outside genes.

Clade	Mean insertion time	Mean insertion time	−log10(p)
	inside genes (Kyr)	outside genes (Kyr)	
Ale	679.7	823.2	95.7
Reina	703.3	914.1	67.2
Alesia	832.0	839.0	0.6
Tork	794.3	911.0	15.5
Ivana	639.1	796.2	72.5
Galadriel	899.8	1051.4	4.6
Ikeros	833.9	940.3	8.5
Tekay	420.0	484.3	25.3
CRM	648.3	742.0	99.0
Ogre	674.1	738.4	8.9
Athila	835.9	1068.5	51.3
TAR	696.6	1007.6	80.6
Bianca	654.2	988.4	34.4
Angela	441.6	791.7	> 300
SIRE	518.1	750.4	> 300
Retand	628.4	916.4	> 300

Insertion times are expressed in thousand years (Kyr). P-values are reported as –log_10_(*p*). Values reported as *>* 300 indicate that –log_10_(*p*) exceeded 300, corresponding to extremely small p-values beyond numerical precision.

Overall, the variation in mean insertion times suggests that different clades have experienced insertion events on different evolutionary timescales, which may have been influenced by biological or environmental factors (**Figure 3**).

To determine whether or not the clade distribution occurs within each class regardless of element type, we applied the chi-square test. There was no significant correlation between the clades and the autonomous type (*X*^2^ = 957.91*,df* = 1095*,p* = 0.99), indicating that clade distribution is independent of the autonomous type. However, for the non-autonomous type, we observed a significant correlation (*X*^2^ = 10,450.18*,df* = 2336*,p <* 0.0001), suggesting that the non-autonomous type influences the clade distribution.

**Figure 4:**
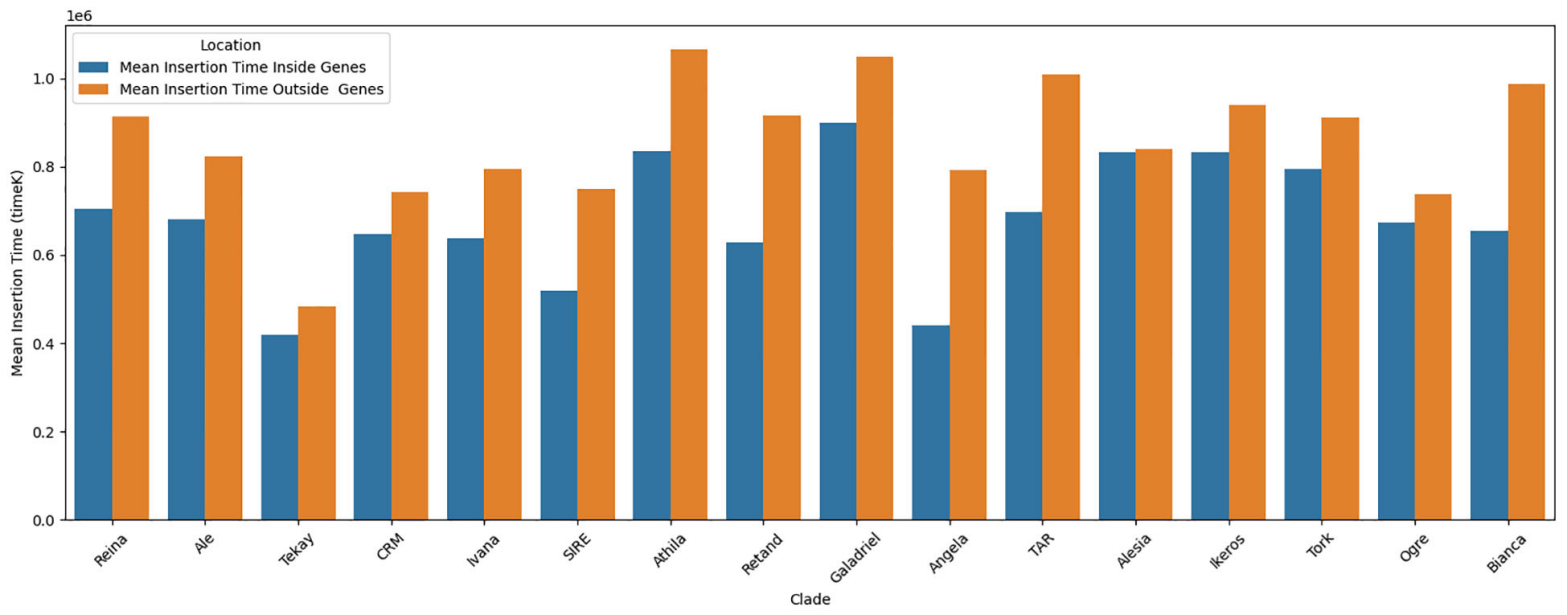
Comparison of mean insertion times (timeK) of LTR-RTs clades inside versus outside gene regions. The analysis highlights clade-specific patterns in insertion timing and genomic targeting behavior.

To characterize the landscape of LTR-RT insertion times across clades, we conducted a comparative analysis to determine whether the insertion times differed significantly between the regions inside and outside the genes for each clade. We applied the Mann-Whitney U test, where data were split by insertion position, with insertion times categorized as either inside or outside the gene. This test examines whether the distributions of these two groups differ significantly. Clades with fewer than two observations in either category were excluded from the analysis. Five clades were present in only a small number of plant species or did not represent defined phylogenetic clades, and therefore lacked sufficient observations in both genic and intergenic categories for meaningful comparison, reducing the dataset from 21 to 16 clades. Of these 16, 93.75% (15 out of 16) showed significant differences in insertion time between genic and intergenic regions (*p* ≤ 0.001), while 6.25% (1 out of 16, Alesia) showed no significant difference (*p >* 0.05). (**Table 1** and **Figure 4**).

## 4 Discussion

### 4.1 Comparative analysis of genome size and LTR-RTs content

The wide variation in LTR-RT content among the 208 analyzed plant species likely reflects differences in transpositional activity, genome defense mechanisms, and removal processes. Species with high LTR-RTs content, such as *Z. mays* and *H. annuus*, may have experienced recent amplification events, whereas species with low content, such as *L. perrieri* and members of the *Gossypium* genus, may exhibit more efficient repression mechanisms and rapid elimination pathways (Baidouri and Panaud, 2013; Staton et al., 2012; Beulé et al., 2015).

The predominance of non-autonomous LTR-RTs (95.57%) across all analyzed genomes suggests widespread degeneration of coding capacity, consistent with long-term evolutionary decay and hostmediated silencing mechanisms. However, this hyperabundance also reflects a parasitic strategy in which non-autonomous elements exploit the transpositional machinery provided by the comparatively scarce (4.43%) autonomous elements (Jiang et al., 2016; Schulman, 2012). The dominance of *Ty3-retrotransposons* over *Copia* elements further supports previous observations that *Ty3-retrotransposons* contribute disproportionately to genome expansion and tend to accumulate in heterochromatic, pericentromeric regions where they escape both homologous recombination and negative selection pressure (Hill et al., 2005; Schulman, 2012; Zhao and Ma, 2013).

Our correlation analyses between genome size and LTR-RT content supports the role of LTR-RTs in genome expansion, consistent with previous studies (Mokhtar et al., 2023b; Yang et al., 2023; Baniaga and Barker, 2019). However, the moderate correlation with percentage values and the presence of notable exceptions indicate that genome size alone does not fully explain LTR-RTs accumulation. Instead, lineagespecific dynamics, including differential amplification, epigenetic silencing, and recombination-mediated removal, likely contribute to this variability (Vitte and Panaud, 2005; Liu et al., 2018; Zhao and Ma, 2013; Dai et al., 2018). Crucially, the quadratic model and the linear model for absolute LTR-RT content reveals a non-linear acceleration threshold in genome bloating, implying that as total plant genome size scales upward, the accumulation of LTR-RTs accelerates rapidly. This pattern may reflect a threshold effect in host–TE dynamics, although we acknowledge that this interpretation extends beyond the correlational data presented here and requires experimental validation.

Nevertheless, the moderate correlation with percentage values (*r* = 0.38) and distinct taxonomic anomalies underscore that genome size alone cannot predict LTR-RT landscapes. For instance, the striking contrast between *P. persica* and *P. dulcis*, which share identical genome sizes (227 Mbp) yet differ sharply in LTR-RT counts, as well as the surprisingly low LTR-RT proportion in the massive genome of *G. arboreum*, demonstrates that lineage-specific dynamics override broad genomic trends. These discrepancies are likely governed by unique balances of differential amplification, highly specific RNAi-directed DNA methylation, and unequal homologous recombination-mediated removal rates across distinct genera (Vitte and Panaud, 2005; Liu et al., 2018; Zhao and Ma, 2013; Dai et al., 2018).

Variation in autonomous element abundance in our analyzed data suggests differences in transpositional potential among species. High autonomous fractions, such as the 16.3% observed in *Z. mays*, may indicate recent amplification events or reduced efficacy of repression mechanisms, whereas species with minimal autonomous representation, such as *P. pruinosa* and *P. coccineus*, may reflect more efficient regulatory systems or prolonged evolutionary quiescence. Collectively, these findings indicate that variation in autonomous LTR-RT abundance represents an important axis of genome evolution, shaping both genome size dynamics and structural diversity across plant lineages (Schulman, 2012; Baidouri and Panaud, 2013; Marsano et al., 2012).

Our findings support the idea that LTR-RTs distribution is influenced by both genomic and population level factors. At the genomic level, LTR-RTs tend to accumulate in heterochromatic regions where recombination is reduced and purifying selection is weaker, while insertions in euchromatin are more frequently removed, especially near genes (Xu and Du, 2014; Peterson-Burch et al., 2004). At the population level, factors such as effective population size and genetic drift may also affect TE accumulation, with smaller populations potentially allowing greater variability and persistence of LTR-RTs (Hosid et al., 2012; Tollis and Boissinot, 2013).

Finally, extensive presence of unclassified LTR-RTs in our analyses such as in *L. rigidum* (33%) and *L. perenne* (30.12%), reveal substantial gaps in current classification frameworks and highlight the potential for discovery (Wicker et al., 2007; Baucom et al., 2009). Overall, these results emphasize the diversity of evolutionary strategies that plants use to regulate LTR-RTs activity. These include lineage-specific amplifications, regulatory repression of autonomous elements, and different LTR-RTs types. Future research should refine classification systems and investigate the role of unknown LTR-RTs to better understand their evolutionary impact.

### 4.2 Statistical analysis of LTR-RTs distribution

#### 4.2.1 Chromosome-wide heterogeneity of LTR-RTs and its biological significance

Our statistical analysis of 208 plant species provides a comprehensive overview of how LTR-RTs are organized within plant genomes. The most striking finding is the near-universal non-random distribution of these elements. Although LTR-RTs densities vary among chromosomes in most species, their spatial arrangement within chromosomes is strongly constrained by genomic architecture rather than determined by random insertion alone. The predominance of significant inter-chromosomal variation observed in our analysis (88.46% of species) indicates that LTR-RTs accumulation patterns are consistent with chromosome-specific structural features rather than uniform genome-wide pressures, consistent with earlier comparative studies (Cossu et al., 2017). Our ANOVA and *Post hoc* LSD results further demonstrate that the genomic load of retrotransposons is unevenly distributed among chromosomes in most plant genomes. This pattern mirrors observations in pear (*Pyrus*) genomes, where LTR-RTs density correlates positively with chromosome size, reflecting the influence of chromosome-specific properties on retrotransposon accumulation (Wang et al., 2025). Similar chromosome-level compartmentalization has been reported in large and structurally complex genomes such as *Z. mays* and *A. tauschii*, where certain chromosomes serve as major repositories for transposable elements (Schnable et al., 2009; Zhao et al., 2016).

The observation that 72.7% of pairwise chromosome comparisons within genomes showed no significant difference in LTR-RT density suggests that many chromosomes are in a state of relative equilibrium regarding retrotransposon load. This homogenization likely reflects shared chromosomal properties such as similar recombination rates, comparable heterochromatin content, and equivalent gene densities, which create similar selective environments for LTR-RT persistence across chromosomes within the same genome. This interpretation aligns with findings in rice and soybean, where chromosomes with similar recombination profiles accumulate comparable retrotransposon densities (Tian et al., 2009, 2012). The presence of significant inter-chromosomal differentiation in a minority of comparisons, alongside broad equilibrium in the majority, therefore reflects a genome organization in which a few chromosomes typically those with the most divergent heterochromatin or recombination landscapes, act as outliers that drive overall statistical heterogeneity, while most chromosomes maintain broadly comparable LTR-RT loads (Lee and Martienssen, 2021).

The striking differences in LTR-RT content between chromosomes, statistically significant in over 88% of the species we examined after FDR correction, are not simply artefacts of our analyses, but rather provide insight into the biological forces that have shaped plant genomes over millions of years. One of the most important of these forces is variation in recombination rate. Chromosomes with extensive pericentromeric and heterochromatic regions, where recombination is suppressed, retain LTR-RTs insertions far more efficiently than recombination-rich chromosomes, because the risk of deleterious ectopic recombination between similar elements is markedly reduced in such domains (Tian et al., 2009; Cossu et al., 2017). This recombination landscape, which differs not only between species but also between chromosomes within the same genome, therefore creates uneven opportunities for LTR-RTs persistence and is consistent with preferential accumulation in recombination-suppressed chromosomal domains while others remain comparatively depleted. Closely linked to this is the distribution of heterochromatin and centromeric DNA, which provides stable safe harbors where LTR-RTs can persist over long evolutionary timescales without exerting strong fitness costs (Lee and Martienssen, 2021). Differences in gene density act as a third major filter, gene-rich chromosomes purge new insertions more rapidly through purifying selection because such insertions are more likely to disrupt regulatory or coding sequences, whereas gene-poor chromosomes tolerate higher densities of intact elements (Hollister and Gaut, 2009). Beyond these structural and selective factors, each chromosome carries its own evolutionary history. Past bursts of LTR-RTs activity, whole genome duplications, and large-scale rearrangements, fusions, fissions, and segmental duplications all leave lasting marks on local repetitive content that can persist long after the events themselves (Schnable et al., 2009; Yang et al., 2023). This historical dimension helps explain why species such as *Z. mays*, *A. tauschii*, and *G. max* exhibit exceptionally strong inter-chromosomal heterogeneity in our analyses, since their genomes have experienced well-documented LTR-RT bursts superimposed on complex polyploid or paleopolyploid backgrounds. In *Z. mays* and *A. tauschii*, this pattern is now confirmed and mechanistically grounded by T2T assemblies revealing chromosome-specific variation in centromeric *Ty3-retrotransposons* content (Chen et al., 2023) and dynamic cycling of LTR-RTs at individual centromeres across evolutionary time (Wlodzimierz et al., 2023). Conversely, species with more uniform distributions, such as *F. vesca*, likely reflect either reduced recent transpositional activity or more efficient host-mediated repression. Taken together, these processes converge to produce the chromosome-level safe harbors pattern, which emerges as a consistent feature of plant genome organization across our dataset; certain chromosomes serve as preferential repositories for LTR-RTs, while others remain transposon-poor. Recognizing these underlying mechanisms reframes inter-chromosomal heterogeneity from a descriptive observation into a meaningful biological readout of the recombination landscape, selective constraints, and evolutionary history that jointly govern plant genome evolution. These mechanistic interpretations are correlational and would require direct chromosome-level covariate modeling to confirm.

#### 4.2.2 Chromosomal patterning of LTR-RTs across plant families

Our multi-test analysis (ANOVA and *post hoc* LSD) reveals that while non-random chromosomal organization of LTR-RTs is a pervasive feature of angiosperms, the intensity of this structuring follows a distinct lineage-specific gradient. Families such as Poaceae, Fagaceae, and Fabaceae exhibit the most pronounced chromosomal bias, with ≥ 90% of ANOVA tests and high proportions of significant pairwise differences (up to 53.25%) indicating intense, localized accumulation. Conversely, lineages such as Ericaceae, Vitaceae, and Cyperaceae show significantly more diffuse LTR-RT distributions, with the lowest proportions of statistical significance, suggesting a more uniform genomic landscape.

These findings indicate that LTR-RT architecture is driven by lineage-specific evolutionary histories rather than neutral accumulation. The high degree of structuring in families like Poaceae likely reflects episodic, stress-induced bursts of LTR-RT amplification. These bursts are often directed toward heterochromatic safe havens to mitigate deleterious impacts on coding regions, a process consistent with established models of subgenome asymmetry and rapid genome evolution (Xu et al., 2010; Liu et al., 2018; Ma et al., 2022).

Finally, the non-random patterns we observed likely reflect historical environmental responses. Stress-induced activation of LTR-RTs, documented in *Populus* and sunflower, can trigger episodic bursts of accumulation (Vangelisti et al., 2019; Mascagni et al., 2020). These bursts, concentrated in heterochromatic “safe havens” to avoid deleterious effects, likely reinforce the strong lineage-specific chromosomal patterns seen in our results. In grasses, where LTR-RTs are highly enriched in heterochromatic regions (Ma et al., 2022), these processes have been particularly influential. Our large-scale analysis confirms that while the mechanisms of LTR-RTs regulation are conserved across angiosperms, the resulting chromosomal architecture is highly dependent on lineage-specific evolutionary history.

#### 4.2.3 Chromosomal distribution patterns of *Copia* and *Ty3-retrotransposons* superfamilies

Our statistical analyses reveal two distinct, complementary dimensions of non-randomness between the major LTR-RT superfamilies. *Ty3-retrotransposons* show stronger spatial structuring at the within chromosome scale. While genome-wide ANOVA detected slightly more frequent heterogeneity in *Copia*, finer-scale analyses revealed the opposite pattern: *Ty3-retrotransposons* elements consistently display more pronounced spatial (within-chromosome) clustering. Chromosome-level chi-square tests yielded significant results for *Ty3-retrotransposons* compared with *Copia*. More strikingly, KS tests showed a higher proportion of significant results for *Ty3-retrotransposons* than for *Copia* (53.29% vs 35.81% after FDR correction), though both values indicate substantial within-chromosome spatial structuring in both superfamilies. Permutation-based variance tests similarly revealed substantial non-random within chromosome clustering in both superfamilies (32.4% *Copia*, 34.8% *Ty3-retrotransposons*), consistent with the ANOVA findings (**Supplementary Table S12–15**), **Supplementary Figure 3**).

These patterns are consistent with established observations across diverse plant genomes. *Ty3retrotransposons* elements are typically more abundant than *Copia* elements and contribute disproportionately to genome size expansion, particularly in large, repeat-rich genomes (Qiu and Ungerer, 2018; Thomas-Bulle et al., 2018; Cao et al., 2024). For example, in rice, *Ty3-retrotransposons* elements are more than four times as abundant as *Copia* elements and are preferentially enriched in pericentromeric regions (Cao et al., 2024); in melon, *Ty3-retrotransposons* elements constitute approximately 26% of the total genome (Ramallo et al., 2008). Such preferential accumulation in recombination-suppressed regions likely underlies the stronger within-chromosome structuring we detect for *Ty3-retrotransposons*.

In contrast, *Copia* elements, although generally less abundant, show greater variability in chromosomal localization and higher transcriptional activity. Elevated *Copia* expression compared to *Ty3-retrotransposons* has been reported in sunflower and *P. trichocarpa*, suggesting more dynamic activity and higher turnover (Qiu and Ungerer, 2018; Cossu et al., 2012; Tetreault and Ungerer, 2016). This is consistent with our observation of slightly more widespread, but spatially less coherent, inter-chromosomal heterogeneity for *Copia*: their distributions appear to be shaped more by recent lineage-specific bursts and local genomic context than by long-term stabilization in heterochromatic domains.

Genome-wide analyses in *S. lycopersicum* similarly demonstrate that *Copia* and *Ty3-retrotransposons* differ substantially in chromosomal dispersion and family-level dynamics, with *Copia* elements displaying broader distribution patterns and higher variability (Paz et al., 2017), consistent with our observation of greater pairwise variability and less pronounced structural organization for *Copia* across our dataset.

Species-level studies further support this divergence. In *Eleocharis*, fluorescence *in situ* hybridization reveals distinct chromosomal localization patterns for *Copia* and *Ty3-retrotransposons* elements, indicating independent evolutionary trajectories and differential accumulation mechanisms. In *Vicia* species, *Ty3retrotransposons* elements dominate the genome, accounting for 18–35% of total DNA, while *Copia* elements remain comparatively underrepresented (Hill et al., 2005). These contrasts mirror our family-level findings, where *Ty3-retrotransposons* within-chromosome structuring is relatively conserved across lineages, while *Copia* distributions are more sensitive to lineage-specific genome organization.

More broadly, the distribution and abundance of LTR-RTs are shaped by chromatin state, recombination rate, and lineage-specific amplification dynamics, all of which contribute to genome organization and evolution Sader et al. (2021); Friesen et al. (2001). These mechanisms likely underlie the distinct spatial patterns observed between the two superfamilies in our study.

From an evolutionary perspective, these distinct spatial signatures highlight a functional trade-off between the two superfamilies. *Ty3-retrotransposons* amplification is a major driver of genome expansion and structural variation (Ungerer et al., 2009; Zuccolo et al., 2008; Neumann et al., 2006), facilitated by long-term retention in heterochromatic domains. In contrast, the more dynamic distribution and higher transcriptional activity of *Copia* elements suggest a greater potential to influence gene regulation and stress responsiveness, despite their lower overall genomic abundance (Qiu and Ungerer, 2018; Ramallo et al., 2008).

Taken together, our results support a general model in which *Ty3-retrotransposons* elements exhibit stronger chromosomal targeting and structural coherence, driven by chromodomain-directed integration into heterochromatic domains, whereas *Copia* elements display greater dispersion and variability, reflecting differences in insertion preferences, evolutionary turnover, and interactions with the chromatin environment. While both superfamilies are non-randomly distributed across plant chromosomes, they show complementary patterns of structuring: *Copia* is more frequently heterogeneous between chromosomes, while *Ty3-retrotransposons* are more strongly structured within chromosomes. This reinforces the view that LTR-RT chromosomal landscapes reflect a balance among superfamily-specific biology, selective constraints, and lineage history.

### 4.3 Evolutionary dynamics of LTR-RTs insertion times

The predominance of younger insertions within genes across 93.75% of clades provides strong evidence for selective filtering of LTR-RTs from functional genomic compartments. For example, in the *Ale* clade, insertions within genes (mean age 680 Kyr) are significantly younger than those outside genes (823 Kyr), indicating elevated turnover in coding regions. Similar patterns were observed for *Angela* and *SIRE*, where genic insertions are substantially younger than intergenic ones (**Table 1**). These trends are consistent with previous observations in *A. thaliana*, where LTR-RTs in gene-rich euchromatic regions are younger and less abundant due to efficient deletion and selective constraints (Mirouze et al., 2009; Hollister and Gaut, 2009; Tenaillon et al., 2011).

The age asymmetry between genic and intergenic regions in our data supports a birth-and-death model of LTR-RTs evolution, in which new insertions arise through transposition bursts and are subsequently filtered by host-mediated selection (Lisch, 2013). Insertions within genes are likely subject to stronger purifying selection due to their potential impact on gene integrity, leading to more rapid turnover. Mechanisms such as unequal homologous recombination and solo-LTR formation contribute to this removal process, particularly in recombination-rich chromosomal arms (Hollister and Gaut, 2009; Zhao and Ma, 2013). In contrast, older elements persist in heterochromatic, low-recombination regions that serve as relative “safe havens” (Bennetzen and Wang, 2014).

Previous studies have shown that LTR-RTs can act as stress-responsive regulatory elements integrated into plant signaling networks. These elements can be activated by various environmental stressors, including heat, drought, oxidative stress, and nutrient deprivation (Papolu et al., 2022). This activation is often mediated by *cis*-regulatory elements within the 5^′^ LTR promoter that interact with stress-responsive transcription factors and hormone signaling pathways such as ethylene, salicylic acid (SA), abscisic acid (ABA), methyl jasmonate (MeJA), and hydrogen peroxide (Salazar et al., 2007). Furthermore, stress conditions may induce chromatin remodeling and histone modifications at LTR-RTs loci, contributing to transcriptional activation and altered expression of nearby genes (Bui and Grandbastien, 2012).

Functionally, LTR-RTs can serve as alternative promoters or enhancers, generating insertional polymorphisms that increase regulatory diversity and adaptive potential (Feschotte, 2008). However, the retention of such insertions appears constrained, as most genic elements remain evolutionarily young; this suggests that only insertions conferring a distinct functional advantage persist over time.

Variation among clades further indicates functional divergence in genomic behavior. Recently active clades such as *Tekay* and *SIRE* may reflect ongoing transpositional activity, although the mechanisms underlying this activity remain unresolved. By contrast, older clades such as *Athila* and *Galadriel* appear more stably embedded within heterochromatic domains. Intermediate clades (e.g., *Ale*, *Angela*, *Ivana*) display balanced dynamics consistent with moderate turnover rates. Collectively, these findings demonstrate that clade-specific insertion dynamics, chromosomal context, recombination, and selective forces jointly shape the temporal and spatial landscape of LTR-RTs in plant genomes.

## 5 Conclusion

This study provides a comprehensive analysis of the chromosomal organization and evolutionary dynamics of LTR-RTs across diverse plant lineages. Using a robust statistical framework, we show that LTR-RTs distributions are non-random and are strongly influenced by large-scale genomic architecture and lineage-specific evolutionary constraints. Our results reveal a fundamental divergence between LTR-RT superfamilies in their contributions to genome structure. *Ty3-retrotransposons* exhibit nominally stronger within-chromosome spatial structuring than *Copia* across most lineages, consistent with chromodomain-directed integration into heterochromatic regions. However, the magnitude of this difference is modest, and effect sizes should be considered alongside significance rates, consistent with long-term accumulation in recombination-suppressed heterochromatic regions. In contrast, *Copia* elements display greater spatial variability and signatures of recent lineage-specific activity, reflecting a more dynamic turnover pattern. This organizational contrast underscores the distinct evolutionary roles these superfamilies play in genome expansion and structural remodeling. Insertion time analyses further indicate that purifying selection contributes to shaping present-day genomic landscapes. The finding that 93.75% of clades harbor significantly younger elements within genes than in intergenic regions supports the rapid removal of deleterious insertions from functional sequences. Collectively, our results suggest that contemporary chromosomal landscapes reflect a dynamic balance between retrotransposon amplification and hostmediated selective filtering. While the core regulatory principles of LTR-RTs activity appear broadly conserved, their genomic outcomes represent lineage-specific evolutionary trajectories. These findings establish a framework for understanding how transposable elements contribute to structural innovation and adaptive potential in plant genomes.

## Data Availability

The original contributions presented in the study are included in the article/**Supplementary Material**. Further inquiries can be directed to the corresponding author.
